# Knee Adduction Moment and Medial Contact Force – Facts about Their Correlation during Gait

**DOI:** 10.1371/journal.pone.0081036

**Published:** 2013-12-02

**Authors:** Ines Kutzner, Adam Trepczynski, Markus O. Heller, Georg Bergmann

**Affiliations:** 1 Julius Wolff Institute, Charité - Universitätsmedizin Berlin, Berlin, Germany; 2 Bioengineering Group, University of Southampton, Highfield, Southampton, United Kingdom; The University of Queensland, Australia

## Abstract

The external knee adduction moment is considered a surrogate measure for the medial tibiofemoral contact force and is commonly used to quantify the load reducing effect of orthopedic interventions. However, only limited and controversial data exist about the correlation between adduction moment and medial force. The objective of this study was to examine whether the adduction moment is indeed a strong predictor for the medial force by determining their correlation during gait. Instrumented knee implants with telemetric data transmission were used to measure tibiofemoral contact forces in nine subjects. Gait analyses were performed simultaneously to the joint load measurements. Skeletal kinematics, as well as the ground reaction forces and inertial parameters, were used as inputs in an inverse dynamics approach to calculate the external knee adduction moment. Linear regression analysis was used to analyze the correlation between adduction moment and medial force for the whole stance phase and separately for the early and late stance phase. Whereas only moderate correlations between adduction moment and medial force were observed throughout the whole stance phase (R^2^ = 0.56) and during the late stance phase (R^2^ = 0.51), a high correlation was observed at the early stance phase (R^2^ = 0.76). Furthermore, the adduction moment was highly correlated to the medial force ratio throughout the whole stance phase (R^2^ = 0.75). These results suggest that the adduction moment is a surrogate measure, well-suited to predicting the medial force ratio throughout the whole stance phase or medial force during the early stance phase. However, particularly during the late stance phase, moderate correlations and high inter-individual variations revealed that the predictive value of the adduction moment is limited. Further analyses are necessary to examine whether a combination of other kinematic, kinetic or neuromuscular factors may lead to a more reliable prediction of the force magnitude.

## Introduction

Osteoarthritis (OA) of the knee joint is a common disease which is accompanied by pain and impaired mobility. Among others, joint loading is one factor that can influence the development and progression of OA [1], [2]. The increased incidence of medial compartment OA is therefore thought to result from higher loading of the medial compartment [3].

A common indirect measure of the medial tibiofemoral contact force (F_med_) is the external knee adduction moment (EAM). This moment is mainly determined by the ground reaction force and its lever arm to the knee joint center. By passing medially to the knee joint center, the force vector creates an adduction moment that is thought to increase the medial compartment load [4]. Studies have shown that the EAM is related to limb alignment [5]–[8], bone mineral density of the proximal tibia [6] and the progression of OA [7], [9]. Due to these findings and a lack of methods for a direct assessment of F_med_, numerous studies have assessed the effect of OA treatments such as laterally wedged shoes, valgus braces and high tibial osteotomies, by analyzing changes of the EAM [7], [10], [11]. However, while there is indirect evidence that the EAM and the actual loads transferred through the medial tibiofemoral compartment are related, the quantitative relationship between EAM and F_med_ is not well established.

Using an analytical computer model, Shelbourne and co-workers compared EAM reduction when walking with braces and wedged shoes to the associated changes of F_med_ and found that the relative reduction of the peak EAM was 2–3 times higher than that of F_med_ [12]. However, accurate consideration of muscle co-contraction and validation of such modeling approaches to determine the tibiofemoral contact forces remain challenging [13], [14]. Using an instrumented knee implant, medial contact forces were measured in one patient in vivo by Zhao and co-workers and compared to the EAM [15]. In this elderly male patient, the study found a moderate correlation between EAM and F_med_ during the stance phase of gait with a coefficient of determination R^2^ that varied between 0.53 and 0.75. Further analyses in the same subject sought to determine whether a reduction of the peak EAM correlates to a reduction of the peak medial force [16], [17]. Using an intervention shoe with greater lateral sole stiffness, the 1^st^ and 2^nd^ peak values of the EAM during the stance phase were found to be reduced by 13% and 22%, respectively, and the reduction of the 1^st^ peak EAM correlated significantly to the reduction of F_med_ (R^2^ = 0.67) [16]. However, this finding could not be confirmed in another study on the same subject in which the gait patterns were modified using walking poles and a ‘medial thrust’ gait [17]. Although the 1^st^ peak EAM was reduced by 32–33%, these reductions did not correspond to reductions of the 1^st^ peak F_med_. These contradictory results highlight the need for further investigation on the predictive value of the EAM for the medial compartment load. Moreover, these parameters were measured in only one subject. The results from more comprehensive in vivo measurements clearly demonstrated substantial inter-individual variation of the tibiofemoral joint contact forces [18]–[20]. Furthermore, we found considerable inter-individual variation in force reductions that resulted from load-altering interventions, such as valgus braces or wedged shoes [21], [22]. To determine whether this inter-subject variability is reflected by the EAM, analyses in a larger sample of subjects are needed.

Therefore, the aim of this study was to examine whether the EAM is a strong predictor for F_med_ by analyzing the correlation between both measures during gait in a larger sample of subjects with telemetric knee implants.

## Materials and Methods

### Instrumented knee implant

Instrumented knee implants with telemetric data transmission were used to measure the tibiofemoral contact forces and moments in vivo [23]. The implants are based on the Innex FIXUC system (Zimmer GmbH, Winterthur, Switzerland), a cruciate sacrificing design with an ultracongruent tibial inlay. The tibial component was modified and equipped with six strain gauges to measure the load-dependent strains in the implant. All signals are sensed and transmitted by a custom-made, inductively powered telemetry circuit [24]. After calibration of each implant, three force components (Fz: axial force, Fx: medio-lateral shear force, Fy: antero-posterior shear force) and three moment components (Mx: flexion-extension, My: varus-valgus, Mz: internal-external rotation) can be measured at a sampling rate of 100 Hz.

The axial force F_z_, is transferred by the medial and lateral compartment. Since the moment M_y_ is caused by the axial force acting eccentrically on the tibia, with a lever arm in the medio-lateral direction, the medial contact force F_med_ can be calculated as follows:


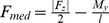
 l: distance between the medial and lateral condyle

Accuracy tests showed that F_med_ can be determined with an error below 3% for forces |F_z_|>1000 N [20]. Therefore, F_med_ was only analyzed during the stance phase of gait and values of |F_z_|>1000 N. Furthermore, the medial force ratio (MR) F_med_/|F_z_|, i.e., the percentage of the axial force that is transferred by the medial compartment, was calculated.

### Ethics statement

This study was approved by the ethics committee of the Charité – Universitätsmedizin Berlin. Nine patients with osteoarthritis provided written informed consent to the procedures and received an instrumented knee implant (Table 1).

**Table 1:** Subject data.

Subject	K1L	K2L	K3R	K4R	K5R	K6L	K7L	K8L	K9L
Sex	m	m	m	f	m	f	f	m	m
Age [years]	64	74	71	67	62	67	76	72	76
Body mass [kg]	100	90	92	102	95	81	69	78	108
Height [m]	1.77	1.71	1.75	1.70	1.75	1.74	1.66	1.74	1.66
Tibio-femoral angle [degree]	3.0 varus	5.0 varus	3.5 varus	4.5 valgus	1.0 varus	4.0 valgus	6.5 varus	4.0 varus	7.0 varus

### Gait analysis

Gait analysis was performed simultaneously with the in vivo force measurement at 27±13 months after surgery. All subjects were walking barefoot at a self-selected comfortable speed on a 10 m long walkway. Ground reaction forces were measured using two 6 degrees of freedom force plates (AMTI, Watertown, MA). Three-dimensional kinematics of the lower limb were tracked at 120 Hz using a 10-camera motion capture system (Vicon, Oxford, UK). A set of 46 reflective markers was used [25]. The complete procedure to determine the skeletal kinematics conditions has been described previously [13]. The segment and joint kinematics, as well as the ground reaction forces and inertial parameters, were used as inputs in an inverse dynamics approach to yield the inter-segmental resultant moments [26], [27]. To calculate the gait velocity, the instances of heel contacts were determined based on the heel marker trajectories.

### Data evaluation

A total of 54 trials (6 trials per subject) were analyzed. Joint contact and ground reaction forces were normalized to bodyweight (%BW) and moments to bodyweight and height (%BWHt).

Regression analysis (SPSS Inc., Chicago, IL, version 18) was used to determine the correlation between EAM and F_med_ and the correlation between EAM and MR. To describe the correlation, coefficients of determination (R^2^) and root-mean-square (RMS) errors between the predicted and observed values of F_med_ were calculated. A linear relationship was assumed between EAM and F_med_ and between EAM and MR, for regression analysis. Because the medial force ratio cannot exceed 100% or fall below 0%, the following arcus tangent function with asymptotic boundaries at 0 and 100% was additionally used for modeling the relationship between EAM and MR:
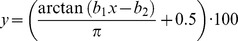



The correlation between EAM and F_med_ was analyzed throughout the whole stance phase (for |F_z_|>1000 N), followed by a separate analysis of the early and late stance phase. The local minimum of the axial ground reaction force at mid stance was taken to distinguish early from late stance phases. Furthermore, the correlations between EAM and F_med_ or MR were analyzed at the two instants of peak medial forces. The correlations were tested for significance considering α = 0.05. A correlation was rated to be good, moderate or poor for a coefficient of determination of R^2^≥0.75, R^2^<0.75 and >0.5, and R^2^≤0.5, respectively.

In order control for the potentially confounding influence of gait velocity and static frontal plane (varus-valgus) limb alignment, further hierarchical multiple regression analyses were performed, where either gait velocity (model i) or alignment (model ii) or both (model iii) were considered as covariates with the EAM as independent and F_med_ as dependent variable.

## Results

**Figure 1:**
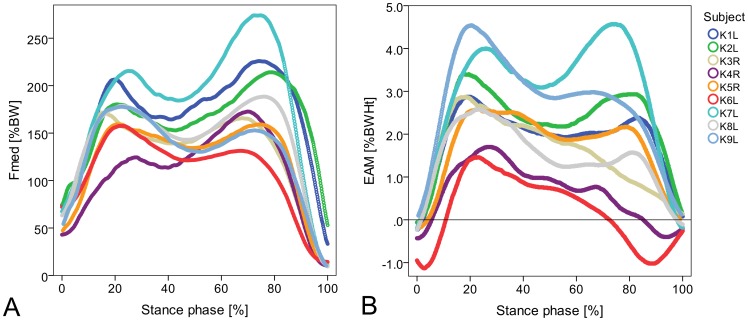
Medial contact forces F_med_ (A) and external adduction moments EAM (B) during the stance phase of gait. Forces are given in % of bodyweight (BW) and moments in %BW times height (Ht). Average curves from 6 repeated trials per subject were calculated using a dynamic time warping procedure [36].

The subjects were walking with an average gait velocity of 1.13 m/s (range: 0.90–1.23 m/s). Clearly discernible peaks of F_med_ occurred at 23±4% and 73±4% of the stance phase of gait (Figure 1A). On average, the peak medial forces during late stance (187±44%BW) were somewhat larger than those observed during early stance (176±27%BW). The pattern of the EAM did not generally resemble the pattern of F_med_. Whereas a first distinct peak of the EAM was observed in all subjects at early stance, only six subjects also exhibited a clearly discernible peak at late stance (Figure 1B). At the time of the 1^st^ and 2^nd^ peak of F_med_, EAM values of 2.9±1.0%BWHt and 2.1±1.3%BWHt were determined, respectively.

### Correlation between external moments and internal forces throughout the stance phase

**Figure 2:**
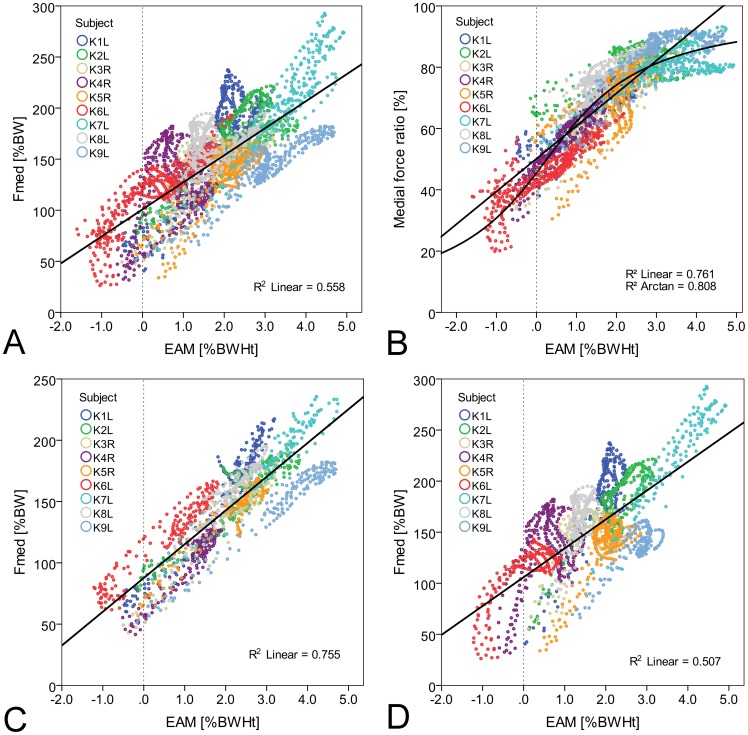
Correlation between external adduction moments EAM and medial contact forces F_med_ during gait. Correlation between EAM and F_med_ during the whole (A), early (C) and late (D) stance phase of gait and correlation between EAM and medial force ratio (B) during the whole stance phase (6 trials per subject).

Throughout the *whole* stance phase (Figure 2A) the coefficient of determination was moderate (R^2^ = 0.56) and the RMS error amounted to 28%BW. When analyzing the correlation between EAM and F_med_ separately for each subject, good correlations were observed in three subjects, moderate correlations in four subjects and poor correlations in the remaining two subjects (Table 2). Moreover, the slopes of the regression lines, the y-intercept values and the RMS errors varied strongly between the subjects.

**Table 2:** Correlations between external adduction moment and medial contact force during gait.

	Whole stance phase				Early stance				Late stance			
Subject	R^2^	RMS error [%BW]	a [%BW]	b	R^2^	RMS error [%BW]	a [%BW]	b	R^2^	RMS error [%BW]	a [%BW]	b
K1L	0.72	24	78	49	0.94	11	76	42	0.76	21	44	74
K2L	0.66	20	89	34	0.85	12	87	28	0.80	15	75	45
K3R	0.54	21	92	27	0.94	9	62	36	0.25	22	108	21
K4R	0.09	34	109	17	0.90	8	57	39	0.25	30	115	36
K5R	0.71	16	61	38	0.90	9	72	32	0.65	17	36	52
K6L	0.78	15	108	32	0.92	9	105	31	0.61	18	106	35
K7L	0.86	17	54	44	0.94	9	77	34	0.91	14	23	55
K8L	0.25	20	125	20	0.90	7	75	38	0.42	18	70	68
K9L	0.90	9	55	28	0.97	6	56	27	0.71	11	34	36
Mean	0.61	20	86	32	0.92	9	74	34	0.60	18	68	47
All	**0.56**	28	101	26	**0.76**	20	88	28	**0.51**	31	106	28

a = y-intercept of the linear regression line, b = slope of the linear regression line.

When analyzing only the *early* stance phase (Figure 2C), high correlations between EAM and F_med_ were observed for individuals (R^2^ = 0.85 to 0.97) and for all subjects combined (R^2^ = 0.76). Especially when analyzing each subject individually RMS errors were small (6–12%BW). Moreover, the y-intercepts and slopes of the individual regression lines exhibited only small deviations across the subjects.

In contrast, only a moderate correlation between EAM and F_med_ for all subjects (R^2^ = 0.51) and high inter-individual variations were observed at the *late* stance phase (Figure 2D). The inter-individual variation is reflected by a wide scatter. For example, at an EAM value of 1.0%BWHt, individual forces F_med_ between 58 and 174%BW were measured. When analyzing the correlation for each individual, three subjects each displayed good, moderate and poor correlations and the RMS errors ranged between 11 and 30%BW.

A good correlation was observed between EAM and MR throughout the whole stance phase (Figure 2B). The coefficient of determination was slightly higher when assuming an arcus tangent function (b_1_ = 0.55, b_2_ = 0.14) instead of a linear function (y = 10.7x+50).

### Correlation between external moments and internal medial peak forces

**Figure 3:**
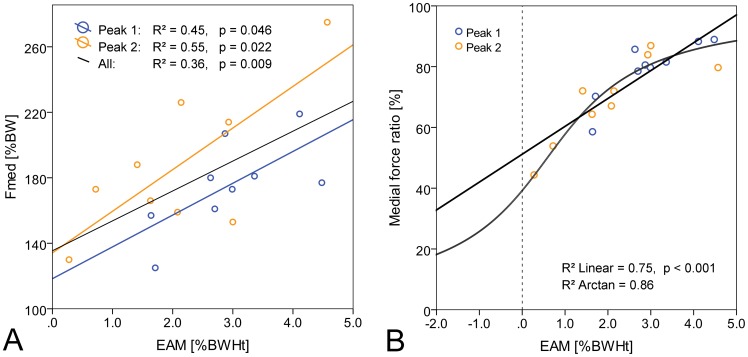
Correlation between peak medial forces F_med_ and external adduction moments EAM. Correlation between peak values of F_med_ (A) or medial force ratios (B) and EAM at early stance (peak 1) and late stance phase (peak 2). Average peak values of nine subjects.

Peak values of F_med_ and the corresponding EAM values were significantly correlated at early and late stance phase (Figure 3A). However, the coefficients of determination were only moderate to poor. A good correlation was observed between EAM and MR, which were both measured at the time of peak F_med_ (Figure 3B). Again, the coefficient of determination was slightly higher when assuming an arcus tangent function (b_1_ = 0.60, b_2_ = 0.35) instead of a linear function (y = 9.2x+51).

### Correlation between external moments and internal medial peak forces considering the co-variates gait velocity and static limb alignment

Both gait velocity and limb alignment were significantly correlated to the peak medial force at the early stance phase (Table 3). When considering both variables (velocity and alignment) the regression model significantly predicted F_med_ with an R^2^ value of 0.76. The hierarchical regression analyses showed no significant change in R^2^ for early stance when considering the EAM and either gait velocity and limb alignment alone or both covariates together were already accounted for (Table 3).

**Table 3:** Correlations between external adduction moment and medial contact force the early stance phase considering the co-variates gait velocity and static frontal plane (varus-valgus) limb alignment.

model	R^2^	adjusted R^2^	change in R^2^	significance of F change	model significance
velocity	0.49	0.42	-	-	0.04
i) velocity, EAM	0.69	0.58	0.20	0.10	0.03
alignment	0.60	0,54	-	-	0.01
ii) alignment, EAM	0.61	0.49	0.01	0.64	0.06
velocity, alignment	0.76	0.68	-	-	0.01
iii) velocity, alignment, EAM	0.77	0.63	0.01	0.72	0.049

At late stance neither velocity nor alignment were significantly correlated to the medial peak force (Table 4). The consideration of the EAM resulted in a significant change in R^2^ beyond the values reached when gait velocity was the only covariate accounted for (change in R^2^: 0.51, p = 0.04). However, none of the regression models accounting for the influence of covariates significantly predicted F_med_ at the late stance phase (p>0.05).

**Table 4:** Correlations between external adduction moment and medial contact force during the late stance phase considering the co-variates gait velocity and static frontal plane (varus-valgus) limb alignment.

model	R^2^	adjusted R^2^	change in R^2^	significance of F change	model significance
velocity	0.05	−0.09	-	-	0.58
i) velocity, EAM	0.56	0.41	0.51	0.04	0.09
alignment	0.27	0.17	-	-	0.15
ii) alignment, EAM	0.58	0.44	0.31	0.08	0.07
velocity, alignment	0.27	0.03	-	-	0.38
iii) velocity, alignment, EAM	0.58	0.33	0.31	0.12	0.19

## Discussion

This study aimed to examine the quantitative relationship between the external knee adduction moment and the medial knee contact force during gait. The results obtained from the analysis of nine subjects with telemetric knee implants confirmed a general correlation between EAM and F_med_ throughout the stance phase of gait and between peak medial forces and corresponding EAM values. However, the variable coefficients of determination as well as high RMS errors reveal the need for a careful interpretation of the EAM.

The correlation of EAM and F_med_ differed substantially between early and late stance phases. Whereas the high linear correlations during the early stance phase suggest that the EAM is a strong predictor for F_med_, moderate to poor correlations during the late stance phase and high inter-individual variance show that this statement cannot be generalized.

In the current literature, controversy exists regarding how the EAM should be analyzed. Whereas many studies focus on the overall peak value of the EAM within the stance phase, others analyze peak EAMs separately at early and late stances or even consider the EAM impulse to provide more information on medial knee joint loading [11], [28]. Considering the inter-individual variance observed in our study, it becomes apparent that the predictive value of the EAM during the late stance is limited. However, the peak medial force during the late stance phase was slightly higher than that observed during the early stance phase. Therefore, force reduction at this later instant may be beneficial or even crucial for reducing pain or slowing down OA progression. As in vivo load measurements have shown, some interventions tended to affect joint contact forces at late stance rather than during early stance [17], [21], [22], [37]. By analyzing only the early stance phase, these important differences in the actual loads transferred at the knee joint cannot be detected.

In addition to the coefficients of determination, the regression lines provide valuable information about the relationship between EAM and F_med_. The slopes and y-intercepts of the linear regression lines reveal that a relative EAM reduction is always higher than the corresponding relative reduction of F_med_. Determined across all subjects, relative EAM reductions within the stance phase were about two times higher than the resulting relative reduction of F_med_. A reduction of the EAM by 10% for example would lead to an average reduction of F_med_ by only 4.6% at early stance and 5.5% at late stance. However, the slopes and y-intercepts of the regression lines differed strongly between the subjects, especially during the late stance phase (Table 2). An EAM reduction of 10% would lead to individual reductions of F_med_ between 3.6 and 5.5% during the early stance phase and 2.7 to 7.6% during the late stance phase. These high inter-individual differences of the regression lines reveal that the same EAM reduction may lead to individually different reductions of F_med_, especially during the late stance phase of gait and indicate that a certain objective, e.g., pain reduction, cannot generally be achieved by a distinct predefined relative EAM reduction.

In the analysis of the relationship between EAM and F_med_ it is important to also consider the potential role of gait velocity as well as static limb alignment. Other studies have already demonstrated that gait velocity mainly influences the peak EAM at the early stance phase [11], [38]. By providing insight into the relationship between gait velocity and F_med_ directly, the results of the current study demonstrate a good correlation between EAM and F_med_ during the early stance phase. However, further hierarchical regression analysis revealed that the EAM did not explain any further variance in F_med_ when gait velocity and static limb alignment were already considered (Table 3). This finding suggests not only that measurement of only two parameters, which are easy to implement clinically (static limb alignment and free walking velocity alone) can already provide a proxy for F_med_ at the early stance phase of gait, but also highlights the need to control for their influence in any study evaluating interventions aiming to modify F_med_. Whilst a substantial amount of the variation in F_med_ expected at the first peak during walking might thus efficiently captured, this is not true for the typically higher forces at the late stance phase. No regression model was identified which significantly predicted peak F_med_ from either EAM or velocity and alignment. Because muscle forces are the major determinants of the loads transferred across the knee, consideration of further analysis techniques such as EMG to assess muscle activation patterns and conditions of co-contraction would appear to be critical to derive improved indirect measures of medial joint loading.

Other than the medial force magnitude, the medio-lateral force distribution across the joint is also an important biomechanical variable. Our results show that the EAM is a stronger predictor for the medial force ratio than for the magnitude of F_med_. These results support the claims of former studies that suggested the EAM represents the relative medio-lateral force distribution rather than the actual force on the medial compartment [4], [29]. This statement was also confirmed by other in vivo load measurements. In a single subject with an instrumented knee implant, a good correlation between EAM and F_med_ or medial force ratio was found [15]. However, similar to our findings, the R^2^ values were higher between EAM and MR than between EAM and F_med_ within the stance phase. This higher correlation between EAM and MR can be explained by considering muscle co-contraction. A change in the level of muscle co-contraction may not influence the medio-lateral force distribution, but may substantially increase the magnitude of F_med_. Therefore, interventions will only be successful in reducing F_med_ by reducing the total joint force or by shifting the force laterally without evoking additional muscle co-contraction. It is possible that interventions that modify the neuromuscular control patterns and the level of co-contraction, but do not change the EAM, can still have the potential to reduce the force magnitudes and can therefore also have a positive effect on OA disease progression, for example. Further investigations on the unloading mechanisms of interventions and gait modifications are necessary to address this issue.

Although our study is unique in that it provides the first analysis of the quantitative relationship between EAM and F_med_ during gait in a larger sample of subjects, the interpretation of our results should also consider potential limitations. While most previous studies concerned with the EAM focused on patients with early to end stage OA, our subjects had total knee replacements. The axial joint force and its medio-lateral distribution are influenced by various interacting factors such as muscle forces, joint kinematics and limb alignment. Following total knee replacement, these factors might be altered. Higher EAM values are more frequent in patients with greater varus alignment or severe OA compared to healthy or less severe OA subjects [5], [30]–[32]. Following total knee replacement, the EAM magnitude can be reduced by correcting varus malalignment [33], [34]. In this study, however, a broad EAM spectrum with individual peak values between 1.6 and 4.6%BWHt was measured and therefore also covered the EAM magnitudes reported for patients with OA [30], [35]. In this study the correlation between EAM and F_med_ was analyzed during free gait, and no interventions aimed at the reduction of either EAM or F_med_ were considered here. The analysis of peak medial forces and corresponding EAM values across all subjects revealed a significant, though poor-to-moderate correlation. However, to analyze the effect of OA treatments, such as laterally wedged shoes, valgus braces or high tibial osteotomies, changes of the peak EAM within one subject are crucial and commonly quantified. In this study, the range of peak EAM values within one subject was too small to determine the effect on intra-individual changes of F_med_.

In the current literature, evidence for the correlation of peak EAM and F_med_ values is limited. In a previous study, the correlation between peak medial forces and peak EAM values was analyzed in a single subject with an instrumented knee implant [16]. In that study a “variable-stiffness shoe” was used as intervention. At early stance, changes of the peak EAM were significantly correlated with changes of peak medial forces. Contradictory results were published in a second study with the same subject performing different gait modifications [17]. To understand the exact mechanisms resulting in either a reduction of the EAM or F_med_ further analyses aiming at the active manipulation of these variables with a larger sample of subjects are necessary. Only though a more detailed understanding of the underlying mechanisms will it become possible to derive and efficiently monitor the outcome of more targeted interventions for reducing F_med_ in a clinical setting.

In conclusion, this study showed that the EAM is a surrogate measure, well-suited to predict the medio-lateral force distribution in the knee joint throughout the stance phase of gait. Although a good correlation between EAM and F_med_ was found during the early stance phase, only moderate correlations and high inter-individual variations during the late stance phase revealed that the predictive value of the EAM is limited. Whether the additional consideration of neuromuscular, kinematic or kinetic factors could help to further improve the prediction of the medial joint contact force remains to be determined.
